# Gender Differences in Mental Health Outcomes before, during, and after the Great Recession

**DOI:** 10.1371/journal.pone.0124103

**Published:** 2015-05-13

**Authors:** Rada K. Dagher, Jie Chen, Stephen B. Thomas

**Affiliations:** Department of Health Services Administration, School of Public Health, University of Maryland, College Park, Maryland, United States of America; Univ of Toledo, UNITED STATES

## Abstract

We examined gender differences in mental health outcomes during and post-recession versus pre-recession. We utilized 2005-2006, 2008-2009, and 2010-2011 data from the Medical Expenditure Panel Survey. Females had lower odds of depression diagnoses during and post-recession and better mental health during the recession, but higher odds of anxiety diagnoses post-recession. Males had lower odds of depression diagnoses and better mental health during and post-recession and lower Kessler 6 scores post-recession. We conducted stratified analyses, which confirmed that the aforementioned findings were consistent across the four different regions of the U.S., by employment status, income and health care utilization. Importantly, we found that the higher odds of anxiety diagnoses among females after the recession were mainly prominent among specific subgroups of females: those who lived in the Northeast or the Midwest, the unemployed, and those with low household income. Gender differences in mental health in association with the economic recession highlight the importance of policymakers taking these differences into consideration when designing economic and social policies to address economic downturns. Future research should examine the reasons behind the decreased depression diagnoses among both genders, and whether they signify decreased mental healthcare utilization or increased social support and more time for exercise and leisure activities.

## Introduction

The United States economy experienced a great recession that officially began in December 2007 and was accompanied by a significant drop in industrial production and real income and a double digit increase in unemployment rates [1, 2]. The literature has shown that economic recessions tend to increase the risk of mental disorder [3, 4] and mental health care utilization [5]. This is of particular importance because mental illnesses top the causes of disability adjusted life years (DALYs) globally and account for 37% of the healthy life years lost from non-communicable diseases [6]. Further, the worldwide cost of mental illness was approximately 2.5 trillion dollars in 2010, projected to increase to over 6 trillion dollars by 2030 [6]. While annual spending on mental healthcare by private health insurance was around 7.0% in 2004–2007, it decreased to 2.1% during 2007–2009 Great Recession in the U.S. [7], which may have negatively impacted people’s ability to access mental health care due to losses in health insurance coverage. To our knowledge, no studies have investigated the relationship between the recent economic recession and population mental health in the U.S. Given the great costs of mental illness, it is important from a mental health policy perspective to identify whether the Great Recession has been associated with worse mental health.

Studies examining the impact of macroeconomic conditions on mental health have yielded mixed findings. A recent review of studies published between 1990 and 2009 found strong evidence supporting adverse effects of job loss on depressive symptoms, suicide, and substance abuse and a moderate positive association between economic contraction and antisocial behavior [8]. In contrast, Ruhm found that a 10% increase in state unemployment rate in a national U.S. sample predicted a 7.3% decrease in non-psychotic mental disorders [9]. Similarly, Viinamaki et al. did not find a significant increase in poor mental health among repeated population samples following Finland’s economic recession (1993–1995) [10].

Many theories have been proposed to explain the associations between economic recessions and mental illness; of the earliest is Durkheim’s seminal work on suicide [11]. He posited that economic changes may result in negative psychological effects that would possibly lead to suicide [11]. Along the same line, Brenner’s empirical work many decades later showed that economic instability resulted in increased rates of psychiatric hospital admissions [12, 13]; either because during tough economic times people experience major stressors in their lives which may provoke new mental illness—“provocation hypothesis”- or economic downturns may uncover already existing cases of mental illness—“uncovering hypothesis” [14]. While each of these different theories is supported by corresponding evidence in the literature, we choose to go with the more mainstream “provocation hypothesis”, especially given the economic stressors that accompanied the recent recession in terms of double digit unemployment rates, loss of real income, and decreased mental health insurance coverage [1, 2]. These harsh economic circumstances constitute major chronic stressors that may have provoked new mental illness [15].

Put in a historical context, economic recessions and periods of high unemployment have been shown to have greater effects on men than women [16]. For example, in the UK, men experienced poorer mental health in 2009 and 2010 than in 2008, while women had no significant changes in their mental health during these years [17]. In the U.S., men have experienced disproportionately more job losses than women in this economic recession, partly because of the greater loss of jobs in the construction and manufacturing sectors than in the service sector [18]; thus, their mental health may be impacted more negatively than women. Moreover, men have lower rates of mental health care utilization than women in general [19]. In fact, research in the U.S. showed that physician visits for treatment of anxiety and/or depression decreased by 7–8% among females and by 25% among males during the economic recession [20]. Thus, we expected differences by gender in the relationship between indicators of the economic recession and mental health outcomes.

Apart from gender differences, the impact of the recession was not spread equally across the nation, with the South and West regions of the U.S. experiencing the largest income losses as measured by the Economic Security Index (ESI) [21]. Moreover, the lowest income groups were hit hardest by the recession, with real household income falling by 12.3% for the lowest income quintile and by 9.6% for the second lowest quintile, from 2007 to 2011 [22]. In addition, the Great Recession has resulted in the longest average unemployment duration since record-keeping for recessions started in 1948 [23]. Research has shown that workers who become unemployed during recessions experience a large decline in lifetime earnings of approximately 19% which is almost double the life-time earnings lost by workers in non-recession periods [24]. Loss of employment is usually also accompanied by loss of employment-based private health insurance which reduces access to care. In fact, research in the U.S. has shown that physician visits for treatment of anxiety or depression significantly decreased during the Great Recession [20]. Thus, we expected that the association of the recession with mental health may potentially vary by U.S. region of residence, income level, employment status, and health services use.

This study examined the relationship between recession indicators (during and after recession versus before) and population mental health separately by gender. We hypothesized that in comparison to the period preceding the recession (1) mental health may decline during the recession and slightly improve after the recession and (2) the recession will be associated with worse mental health among males than among females and that these gender differences in mental health will be ameliorated in the aftermath of the recession. We also conducted stratified analyses by U.S. region, employment status, income, and health services use, given the potential differential association of the recession with mental health across these different subgroups of people.

## Materials and Methods

### Data

This study utilized 2005–2006, 2008–2009, and 2010–2011 cross-sectional data—designating the pre-recession, during recession, and post-recession periods—from the Medical Expenditure Panel Survey (MEPS), a nationally representative survey of the U.S. non-institutionalized civilian population [25]. MEPS utilizes a complex national probability sampling methodology, which includes stratification, clustering and oversampling of certain population subgroups [26]. MEPS oversamples minority populations such as African Americans, Hispanics, and Asians, and policy-relevant subgroups such as low income respondents. MEPS’s respondents, on average, are interviewed five times over two and a half years using computer-assisted personal interviewing technology. The MEPS response rate ranges between 57 and 78% [27]. The MEPS consolidated file includes information on patients’ socio-demographic characteristics and mental health measures, including self-reported mental health, the 12-item Short Form Mental Health Summary (SF-12 MCS) measure, and the Kessler 6 (K6) scale of non-specific psychological distress. The MEPS medical condition files contain data on respondents’ chronic diseases self-reported by patients and then coded by professional coders to fully-specified ICD-9-CM codes. The MEPS medical condition files were linked to the consolidated files to get a comprehensive dataset on patients’ demographics (e.g. gender, race/ethnicity), socioeconomic status (e.g. education, income), and mental health outcomes.

The study population included all adults aged 18 to 64 years old. There were no exclusion criteria. The study weighted sample included a total of 46,408 females and a total of 34,905 males. The study was approved by the Institutional Review Boards at the University of Maryland. Our analysis is based on a public data set. All the patient information was anonymized and de-identified prior to analysis.

### Variables

#### Outcome Variables

We had five mental health outcome variables: depression diagnosis, anxiety diagnosis, self-reported mental health, SF-12 MCS score, and K6 score. Using the ICD9 codes provided in the MEPS dataset, we were able to identify whether the respondent had been diagnosed with depression (ICD9 = 311) or anxiety (ICD9 = 300) disorders and coded each as dummy variables. Depression and anxiety were self-reported and validated by contacting respondents’ physicians. While depression and anxiety disorders often occur concurrently as they both share a common dimension, negative affect, they differ in that depression lacks positive affect and physiological hyperarousal is unique to anxiety [28]. Self-rated mental health was measured using the question, “In general, would you say that your mental health is excellent, very good, good, fair, or poor?” The responses ranged from 1 (poor) to 5 (excellent). Self-rated mental health in the MEPS has been validated and found to be related to psychological distress symptoms; however, it cannot be used as a substitute for them [29]. The SF-12 MCS measures general mental health that does not target a specific age or disease group and is internationally recognized for its validity and reliability [30]. In the MEPS dataset, the SF-12 MCS has been found to have high internal consistency reliability (α = 0.82) and high predictive validity in relation to cognitive limitations [31]. It constitutes of 6 items that address 4 content domains of general mental health: social function (1 item), mental health (2 items), vitality (1 item), and role limitations due to emotional health (2 items) [30]. The items were summed to a score that ranges from 0 to 100 and higher scores denote better mental health. The “K6” scale developed by Kessler et al. includes six mental health-related questions that assess the person’s non-specific psychological distress during the past 30 days [32]. These include: feeling nervous; hopeless; restless or fidgety; so sad that nothing could cheer the person up; everything was an effort; and worthless. The items were summed to a score that ranges from 0 to 24 and higher scores denote a greater tendency towards mental disability. In the MEPS dataset, factor analyses that combined the 20 items from the SF-12 MCS, K6, the 2-item Patient Health Questionnaire (PHQ-2), and self-reported mental health, showed that all items of the K6 scale loaded strongly on the factor “mental health” [29].

#### Independent Variables

The key explanatory variables included two dummy variables designating the survey period during the recession (2008–2009) and post-recession (2010–2011), with the reference category being the pre-recession period (2005–2006). Our analyses examined mental health outcomes for females and males separately.

Based on a priori causal assumptions, several covariates pertaining to demographic, socioeconomic, and health factors that could confound the relationship between the economic recession and mental health outcomes were included as control variables. These variables have been widely used in the literature [33, 34]. The covariates we controlled included age, race/ethnicity (Hispanic, African American, Other Race versus White), education (high school graduate versus no high school degree), marital status (married versus not), employment status (unemployed versus employed), family income (below 100% Federal Poverty Level (FPL), 100%-200% FPL, versus >200% FPL), health insurance status [private insurance, public insurance (Medicaid and/or Medicare), versus uninsured], having a usual source of care or not, interviewed in English versus Spanish, living in an urban versus a rural area, and US census region (West, Midwest, South, versus Northeast). We also controlled for the presence of the following chronic diseases: stroke, acute myocardial infarction (AMI), hyperlipidemia, hypothyroidism, and hypertension, as studies show that mental health disorders are often comorbid with physical chronic diseases [35].

### Analysis

We used STATA 12.0 (StataCorp LP, College Station, TX) to perform all the analyses, while accounting for the complex sampling design of the MEPS. We corrected the standard errors due to clustering within strata and the primary sampling unit, and we applied sampling weights to produce estimates that take into account the MEPS complex design, unequal probabilities of selection, and the non-response rate of the survey. We conducted Chow tests to test whether our regression models for each of the mental health outcomes differed for males versus females [36]. Weighted statistics of the covariates were first summarized for males and females and compared before, during, and after the economic recession, using t-tests and Bonferroni comparisons. Multivariate logistic regression models were conducted to examine the association between the recession and two categorical mental health outcomes: depression diagnosis and anxiety diagnosis, controlling for the covariates listed above. Ordinary least squares regressions were utilized to examine the association between the recession and three continuous mental health outcomes: the self-reported mental health, the SF-12 MCS score, and the Kessler 6 (K6) score. These regressions were conducted separately by gender in accordance with the Chow test findings. Moreover, we conducted sensitivity analyses to test the robustness of our results. Specifically, we conducted stratified analyses by U.S. region, employment status, income, and among respondents who had received mental health treatment. We used the same control variables in the stratified analyses as in the full models (except for the stratified variable in question). In all our analyses we used a p-value cutoff of 0.05 to indicate significance.

## Results

Due to the potential of our conceptual model to apply differently for males and females, we conducted a Chow test for each of the mental health outcome variables. The Chow test results showed significant differences between regression coefficients for males and females in four of the five outcome variables (diagnosis of depression: F = 505.27, p<0.001; diagnosis of anxiety: F = 372.26, p<0.001; self reported mental health: F = 0.59, p = 0.56; the SF-12 MCS, F = 187.82, p<0.001; the Kessler 6: F = 51.56, p<0.001). Thus, the association of the recession with mental health outcomes differs by whether a person is a male or a female.

The weighted summary statistics of our sample before, during, and after the recession are presented in Table 1 separately by gender. We utilized t-tests to compare the means during and after the recession to those before the recession. In addition, we used the Bonferroni adjustment for multiple comparisons to compare the three time periods altogether for all the categorical variables.

**Table 1:** Weighted summary statistics of the sample before, during, and after the recession^†^.

		Females				Males		
Timing in relation to the Great Recession	Before n = 15454	During n = 15975	After n = 14979	Bonferroni Test	Before n = 11499	During n = 12061	After n = 11345	Bonferroni Test
	Mean	Mean	Mean	*P*	Mean	Mean	Mean	*P*
Depression Diagnosis	0.14	0.14	0.14	NS	0.09	0.08	0.09	<0.05
Anxiety Diagnosis	0.10	0.11	0.12***	NS	0.06	0.06	0.06	NS
Self-Reported Mental Health Score	3.93	3.94	3.92		3.97	3.99	3.96	
MCS Score	49.39	49.11	49.28		51.14	51.08	51.07	
Kessler Index Score	3.94	4.01	3.99		3.42	3.49	3.43	
Race				<0.01				<0.001
White	0.70	0.70	0.68		0.73	0.71	0.71	
Hispanic	0.12	0.12	0.13		0.12	0.13	0.13	
African American	0.12	0.12	0.12		0.09	0.09	0.10	
Other race	0.06	0.06	0.07		0.06	0.06	0.06	
Age	41.04	41.44*	41.68**	<0.01	41.65	41.88	42.13*	<0.05
Married	0.53	0.53	0.52	NS	0.54	0.54	0.52*	NS
No High School	0.17	0.17	0.15*	NS	0.20	0.20*	0.17**	NS
Unemployed	0.22	0.23	0.24***	NS	0.12	0.13**	0.16***	NS
Have a Usual Source of Care	0.83	0.82*	0.82	NS	0.75	0.73	0.75	NS
Insurance Status				NS				NS
Uninsured	0.12	0.13*	0.13		0.16	0.17	0.16	
Public Health insurance	0.12	0.13	0.14***		0.07	0.08	0.09***	
Private Health insurance	0.76	0.74**	0.74**		0.78	0.76*	0.75***	
Interviewed in English	0.95	0.94	0.95	NS	0.94	0.94	0.94	NS
Family Income				NS				NS
Family income <100% FPL	0.12	0.13	0.15***		0.08	0.10**	0.11**	
Family income 100% -200% FPL	0.15	0.16	0.17*		0.14	0.15	0.15***	
Family income >200% FPL	0.73	0.71	0.68***		0.78	0.75*	0.74***	
Stroke	0.001	0.0006*	0.0007	NS	0.001	0.0004*	0.0008	NS
AMI	0.002	0.01***	0.01***	<0.001	0.01	0.02***	0.02***	<0.001
Hyperlipidemia	0.10	0.17***	0.16***	<0.001	0.14	0.22	0.22***	<0.001
Hypothyroidism	0.05	0.05	0.05	NS	0.01	0.01	0.01	NS
Hypertension	0.17	0.20***	0.21***	<0.05	0.19	0.24***	0.26***	<0.05
Urban Residence	0.84	0.84	0.85	NS	0.83	0.84	0.84	<0.05
U.S. Census Region				NS				NS
Northeast	0.18	0.18	0.18		0.18	0.18	0.18	
Midwest	0.23	0.23	0.23		0.24	0.23	0.24	
South	0.36	0.36	0.37		0.35	0.35	0.36	
West	0.22	0.22	0.23		0.23	0.24	0.23	

^†^ Starred P-values represent comparisons of means during and after the recession compared to pre-recession. The two columns of p-values represent the results of Bonferroni tests, which are used to test the significant associations of the “during, before, and after” recession periods with the categorical variables for females and males, respectively (NS = non-significant).

* p ≤ 0.05;

** p ≤ 0.01;

*** p ≤ 0.001

### Female summary statistics

Among the five mental health outcomes for females, the only significant finding was that females were slightly more likely to be diagnosed with anxiety during and after the recession (11%; 12%) than before the recession (10%). The characteristics of the female samples during and after the recession did not differ from those before the recession in terms of racial/ethnic composition, marital status, language of the interview, presence of hypothyroidism, urban versus rural residence, or by geographic location. The sample of females during the recession was older (41.44 vs. 41.04), less likely to have a usual source of care (82% vs. 83%), more likely to be uninsured (13% vs. 12%), less likely to have private insurance (74% vs. 76%), less likely to have a stroke (0.06% vs. 0.1%), more likely to have AMI (1% vs. 0.2%), more likely to have hyperlipidemia(17% vs. 10%), and more likely to have hypertension (20% vs. 17%) than the sample of females before the recession. The sample of females after the recession was older (41.68 vs. 41.04), more likely to be unemployed (24% vs. 22%), more likely to have public insurance (14% vs. 12%), less likely to have private insurance (74% vs. 76%), and more likely to be below 100% FPL (15% vs.12%) than the sample of females before the recession.

Bonferroni comparisons for females showed no significant differences across the three time periods by depression diagnoses, or anxiety diagnoses. However, they showed significant differences by racial/ethnic composition and presence of AMI, hyperlipidemia, and hypertension.

### Male summary statistics

The five mental health outcomes for males did not significantly differ during and after the recession compared with the period before the recession. The characteristics of the male samples during and after the recession did not differ from those before the recession in terms of racial/ethnic composition, language of the interview, usual source of care, the percentage of uninsured, urban versus rural residence, or by geographic location. The sample of males during the recession was more likely to be unemployed (13% vs. 12%), less likely to have private insurance (76% vs. 78%), more likely to be below 100% FPL (10% vs. 8%), less likely to be above 200% FPL (75% vs. 78%), less likely to have stroke (0.04% vs. 0.1%), but more likely to AMI (2% vs. 1%), and hypertension (24% vs. 19%) than the sample of males before the recession. The sample of males after the recession was older (42.13 vs. 41.65), less likely to be married (52% vs. 54%), more likely to be unemployed (16% vs. 12%), more likely to have public insurance (9% vs. 7%), less likely to have private insurance (75% vs. 78%), more likely to be below 100% FPL (11% vs. 8%), more likely to meet 100%-200% FPL (15% vs.14%), less likely to be above 200% FPL (74% vs. 78%), and more likely to have AMI (2% vs. 1%), hyperlipidemia (22% vs. 14%), and hypertension (26% vs. 19%) than the sample of males before the recession.

Bonferroni comparisons for males showed significant differences across the three time periods by depression diagnoses, but not by anxiety diagnoses. Moreover, they showed significant differences by racial/ethnic composition, presence of AMI, hyperlipidemia, and hypertension, and urban versus rural residence.

### Regression results for females

As shown in Table 2, multivariate regression results showed that females had lower odds of being diagnosed with depression during the recession and after the recession compared to the period prior to the recession. In contrast, females had higher odds of being diagnosed with anxiety after the recession compared to pre-recession. Another way to present findings on anxiety and depression diagnoses is to examine marginal effects (ME) of the recession on these two outcomes. Results showed that females had 1% more diagnoses of anxiety after the recession (ME = 0.01) than before the recession. Moreover, females had 2% less depression during the recession (ME = -0.02) and 1% less depression diagnoses after the recession (ME = -0.01), compared to pre-recession. In addition, females had better self-reported mental health scores during the recession compared to pre-recession.

**Table 2:** Multivariate analyses of the associations between recession indicators (during and after) and mental health outcomes for females.

	Depression Diagnosis (N = 50,557)		Anxiety Diagnosis (N = 50,557)		Self-Reported Mental Health Score (N = 50,484)		MCS Score (N = 47,669)		Kessler Index Score (N = 46,719)	
	OR	95% CI	OR	95% CI	Coef.	95% CI	Coef.	95% CI	Coef.	95% CI
Before Recession	ref		ref		ref		ref		ref	
During Recession	0.86***	0.78, 0.95	1.04	0.94, 1.16	0.04*	0.00, 0.07	-0.09	-0.39, 0.21	-0.07	-0.20, 0.06
After Recession	0.89*	0.80, 0.98	1.17***	1.05, 1.30	0.03	0.00, 0.07	0.15	-0.14, 0.44	-0.12	-0.26, 0.01
Hispanic	0.68***	0.59, 0.79	0.56***	0.48, 0.66	0.03	-0.02, 0.07	0.70***	0.24, 1.17	-0.18	-0.38, 0.03
Black	0.42***	0.37, 0.47	0.30***	0.26, 0.34	0.12***	0.08, 0.16	1.72***	1.34, 2.10	-0.81***	-0.98, -0.64
Other Race	0.58***	0.48, 0.71	0.50***	0.40, 0.62	0.05	0.00, 0.10	0.94***	0.46, 1.42	-0.15	-0.36, 0.07
Age	1.01***	1.01, 1.02	1.00	1.00, 1.01	-0.01***	-0.01, -0.01	0.03***	0.02, 0.04	0.00	0.00, 0.00
Married	0.65***	0.60, 0.71	0.77***	0.68, 0.86	0.10***	0.08, 0.13	1.29***	1.01, 1.57	-0.65***	-0.77, -0.54
No High School	0.94	0.85, 1.05	0.94	0.82, 1.08	-0.20***	-0.23, -0.16	-0.78***	-1.14, -0.41	0.65***	0.48, 0.83
Unemployed	1.42***	1.29, 1.56	1.38***	1.24, 1.53	-0.19***	-0.22, -0.16	-1.66***	-1.98, -1.34	1.13***	0.98, 1.28
Have Usual Source of Care	1.51***	1.35, 1.69	1.57***	1.38, 1.79	-0.06***	-0.08, -0.03	-0.31*	-0.60, -0.01	0.14*	0.01, 0.26
Public Insurance	1.66***	1.46, 1.89	1.70***	1.46, 1.99	-0.17***	-0.21, -0.12	-1.89***	-2.40, -1.38	1.03***	0.79, 1.28
Private Insurance	0.96	0.85, 1.09	1.01	0.87, 1.18	0.17***	0.13, 0.21	1.53***	1.13, 1.93	-0.77***	-0.96, -0.59
Interviewed in English	1.39***	1.16, 1.65	1.98***	1.58, 2.49	-0.07**	-0.13, -0.02	-0.71**	-1.26, -0.17	0.70***	0.43, 0.98
Family Income <100% FPL	1.37***	1.23, 1.54	1.32***	1.15, 1.51	-0.22***	-0.26, -0.18	-2.30***	-2.76, -1.83	1.24***	1.03, 1.46
Family Income 100–200% FPL	1.31***	1.18, 1.46	1.12*	1.00, 1.24	-0.17***	-0.20, -0.14	-1.59***	-1.92, -1.26	0.82***	0.66, 0.99
Stroke	2.40	0.90, 6.38	0.68	0.26, 1.80	-0.64*	-1.23, -0.05	-5.02***	-8.28, -1.76	1.86**	0.43, 3.28
AMI	1.24	0.88, 1.74	1.36	0.92, 2.00	-0.24**	-0.44, -0.05	-3.34***	-5.22, -1.47	1.99***	0.93, 3.04
Hyperlipidemia	1.54***	1.38, 1.73	1.41***	1.24, 1.61	-0.13***	-0.16, -0.09	-1.06***	-1.47, -0.65	0.61***	0.43, 0.79
Hypothyroidism	1.57***	1.33, 1.84	1.28**	1.06, 1.55	-0.11***	-0.17, -0.06	-0.63*	-1.21, -0.06	0.25	-0.01, 0.51
Hypertension	1.24***	1.12, 1.36	1.29***	1.15, 1.45	-0.16***	-0.19, -0.12	-0.90***	-1.25, -0.54	0.64***	0.47, 0.81
Urban Residence	1.08	0.96, 1.21	1.20**	1.04, 1.39	0.04	0.00, 0.09	0.09	-0.30, 0.48	-0.09	-0.26, 0.07
Midwest	1.29***	1.14, 1.46	1.10	.095, 1.28	-0.01	-0.05, 0.04	-0.10	-0.56, 0.36	0.20	-0.02, 0.42
South	1.16*	1.02, 1.32	1.11	0.95, 1.29	0.04	0.00, 0.09	-0.46*	-0.90, -0.02	0.31***	0.10, 0.52
West	1.22***	1.07, 1.39	1.06	0.91, 1.23	0.05	0.00, 0.10	-0.64**	-1.09, -0.19	0.32***	0.10, 0.54
_constant	0.04***	0.03, 0.05	0.03***	0.02, 0.04	4.29***	4.19, 4.39	48.55***	47.51, 49.59	3.16***	2.71, 3.61

* p ≤ 0.05;

** p ≤ 0.01;

*** p ≤ 0.001

We conducted stratified analyses to see whether these findings are consistent across the four different regions of the U.S. (Tables 3 and 4). We found that females in the Northeast and Midwest regions had higher odds of anxiety after the recession than before the recession. Moreover, females in the South region had lower odds of being diagnosed with depression during and after the recession compared to pre-recession, and those living in the Midwest had lower odds of depression during the recession compared to pre-recession. Females in the South region also had better self-reported mental health during and after the recession compared to pre-recession, and those living in the West region had better self-reported mental health after the recession. In addition, females living in the South had better mental health summary scores and lower scores on the Kessler scale (i.e. lower tendency towards depression and mental disability) after the recession compared to pre-recession.

**Table 3:** Stratified multivariate analyses of recession indicators (during and after) and mental health diagnoses for females.

	Depression Diagnosis (N = 50557) ref.- Before Recession				Anxiety Diagnosis (N = 50557) ref.- Before Recession			
	During		After		During		After	
	OR	95% CI	OR	95% CI	OR	95% CI	OR	95% CI
Females Stratified by Region								
Northeast	0.88	0.71, 1.10	1.11	0.89, 1.40	1.25	0.98, 1.59	1.43*	1.06, 1.92
Midwest	0.83	0.69, 1.00	0.89	0.73, 1.08	1.13	0.92, 1.39	1.53***	1.27, 1.83
South	0.81*	0.68, 0.96	0.82*	0.69, 0.97	0.88	0.72, 1.06	0.98	0.81, 1.19
West	0.95	0.80, 1.14	0.84	0.68, 1.02	1.10	0.90, 1.34	0.97	0.79, 1.19
Females Stratified by Family Income								
<100% FPL	0.82*	0.68, 0.98	0.88	0.74, 1.06	1.05	0.83, 1.32	1.41**	1.12, 1.76
100–200% FPL	0.92	0.77, 1.10	0.82*	0.67, 0.99	1.06	0.84, 1.33	1.22	0.97, 1.53
>200% FPL	0.85*	0.75, 0.97	0.90	0.79, 1.03	1.05	0.92, 1.19	1.11	0.97, 1.26
Females Stratified by Employment								
Employed	0.89	0.79, 1.00	0.90	0.80, 1.01	1.04	0.91, 1.18	1.13	1.00, 1.29
Unemployed	0.81*	0.68, 0.96	0.85	0.72, 1.00	1.05	0.86, 1.28	1.25*	1.03, 1.51

* p ≤ 0.05;

** p ≤ 0.01;

*** p ≤ 0.001

**Table 4:** Stratified multivariate analyses of recession indicators (during and after) and mental health indicators for females.

	Self-Reported Mental Health Score (N = 50484) ref.- Before Recession				MCS Score (N = 47669) ref.- Before Recession				Kessler Index Score (N = 46719) ref.- Before Recession			
	During		After		During		After		During		After	
	Coef.	95% CI	Coef.	95% CI	Coef.	95% CI	Coef.	95% CI	Coef.	95% CI	Coef.	95% CI
Females Stratified by Region												
Northeast	-0.02	-0.10, 0.07	-0.06	-0.14, 0.01	0.09	-0.59, 0.78	0.26	-0.56, 1.08	-0.09	-0.40, 0.22	-0.14	-0.52, 0.24
Midwest	-0.03	-0.09, 0.03	-0.03	-0.10, 0.04	-0.46	-1.13, 0.21	-0.37	-0.99, 0.24	0.05	-0.22, 0.33	0.03	-0.24, 0.29
South	0.10**	0.04, 0.16	0.09*	0.02, 0.16	0.17	-0.29, 0.64	0.52*	0.06, 0.98	-0.20	-0.41, 0.01	-0.23*	-0.44, -0.01
West	0.05	-0.01, 0.12	0.08*	0.01, 0.16	-0.22	-0.84, 0.41	0.04	-0.55, 0.62	-0.01	-0.28, 0.25	-0.11	-0.38, 0.16
Females Stratified by Family Income												
<100% FPL	0.05	-0.03, 0.12	0.06	-0.01, 0.13	0.21	-0.58, 1.00	0.25	-0.55, 1.05	-0.23	-0.61, 0.15	-0.10	-0.50, 0.29
100–200% FPL	0.06	0.00, 0.13	0.05	-0.02, 0.13	-0.08	-0.78, 0.62	0.59	-0.04, 1.22	-0.06	-0.39, 0.26	-0.34*	-0.65, -0.02
>200% FPL	0.03	0.00, 0.07	0.03	-0.01, 0.06	-0.10	-0.45, 0.25	0.07	-0.27, 0.40	-0.06	-0.20, 0.07	-0.09	-0.23, 0.05
Females Stratified by Employment												
Employed	0.04	0.00, 0.07	0.03	-0.01, 0.07	-0.18	-0.52, 0.16	0.12	-0.19, 0.44	-0.04	-0.17, 0.10	-0.11	-0.25, 0.02
Unemployed	0.04	-0.02, 0.11	0.05	-0.02, 0.12	0.20	-0.46, 0.85	0.24	-0.42, 0.89	-0.18	-0.51, 0.14	-0.15	-0.47, 0.17
Females Stratified by Health Service Use												
Users	0.03	-0.05, 0.10	0.03	-0.04, 0.11	0.00	-0.01, 0.00	0.00	-0.01, 0.01	0.23	-0.18, 0.63	0.08	-0.34, 0.50
Non-users	-0.08	-0.17, 0.03	-0.12*	-0.24, -0.01	-0.01	-0.02, 0.00	-0.01	-0.02, 0.00	0.33	-0.19, 0.85	0.30	-0.25, 0.85

* p ≤ 0.05;

** p ≤ 0.01;

*** p ≤ 0.001

We also explored whether our findings differed by employment status by conducting separate analyses on unemployed versus employed females (Tables 3 and 4). We found that among the unemployed, females had lower odds of being diagnosed with depression during and after the recession compared to pre-recession, and had higher odds of being diagnosed with anxiety after the recession compared to pre-recession. On the other hand, among the employed, females had lower odds of being diagnosed with depression and better self-reported mental health during the recession compared to pre-recession.

Stratification analyses by income among females showed that people with low household income had lower odds of depression diagnoses during the recession and higher odds of anxiety after the recession compared to pre-recession (Tables 3 and 4). People with middle household income had lower odds of depression diagnoses and lower scores on the Kessler scale after the recession compared to before the recession. Finally, people with high household income had lower odds of depression diagnoses during the recession compared to pre-recession.

We also conducted stratified analyses on health care utilization among those who were diagnosed with anxiety or depression (Table 4). We defined users as those people who used at least one service. We found that among females, non-users of health services had worse mental health summary scores during the recession and worse self-reported mental health after the recession, compared to pre-recession. However, female users of health services showed no differences in mental health outcomes.

### Regression results for males

As shown in Table 5, multivariate regression results showed that males had lower odds of being diagnosed with depression. Another way to present findings on depression diagnoses is to examine marginal effects (ME) of the recession on this outcome. Results showed that males had 2% less depression diagnoses during the recession (ME = -0.02) and 1% less depression diagnoses after the recession (ME = -0.01), compared to pre-recession. Moreover, males had better self-reported mental health during the recession and after the recession compared to the period prior to the recession. In addition, males had lower scores on the Kessler sale after the recession compared to pre-recession (i.e. lower tendency towards depression and mental disability).

**Table 5:** Multivariate analyses of the associations between recession indicators (during and after) and mental health outcomes for males.

	Depression Diagnosis (N = 38,670)		Anxiety Diagnosis (N = 38,670)		Self-Reported Mental Health Score (N = 38,592)		MCS Score (N = 35,713)		Kessler Index Score (N = 35,164)	
	OR	95% CI	OR	95% CI	Coef.	95% CI	Coef.	95% CI	Coef.	95% CI
Before Recession	ref		ref		ref		ref		ref	
During Recession	0.79***	0.69, 0.90	0.98	0.83, 1.16	0.05***	0.02, 0.09	0.10	-0.23, 0.44	-0.04	-0.18, 0.11
After Recession	0.87*	0.77, 0.99	1.01	0.86, 1.19	0.04*	0.00, 0.07	0.27	-0.08, 0.62	-0.20**	-0.34, -0.05
Hispanic	0.71***	0.59, 0.85	0.61***	0.46, 0.80	0.00	-0.05, 0.05	0.80***	0.33, 1.27	-0.30**	-0.53, -0.07
African American	0.37***	0.31, 0.45	0.31***	0.24, 0.39	0.08***	0.04, 0.12	1.78***	1.38, 2.18	-0.79***	-0.96, -0.61
Other Race	0.62***	0.49, 0.78	0.48***	0.36, 0.63	0.04	-0.01, 0.09	0.66**	0.13, 1.19	-0.23	-0.48, 0.01
Age	1.01**	1.00, 1.01	0.99*	0.99, 1.00	-0.01***	-0.01, -0.01	0.01	0.00, 0.02	0.00	-0.01, 0.00
Married	0.71***	0.64, 0.80	0.75***	0.65, 0.87	0.09***	0.07, 0.12	1.06***	0.78, 1.35	-0.50***	-0.63, -0.37
No High School	1.01	0.88, 1.16	0.87	0.74, 1.02	-0.20***	-0.23, -0.16	-0.62***	-1.01, -0.23	0.43***	0.25, 0.62
Unemployed	1.98***	1.70, 2.30	1.98***	1.69, 2.33	-0.34***	-0.39, -0.28	-3.17***	-3.70, -2.64	1.92***	1.68, 2.16
Have Usual Source of Care	1.36***	1.19, 1.55	1.89***	1.61, 2.22	-0.02	-0.05, 0.01	-0.23	-0.52, 0.07	0.10	-0.03, 0.23
Public Insurance	1.54***	1.26, 1.88	1.58***	1.26, 1.99	-0.27***	-0.33, -0.21	-2.43***	-3.09, -1.77	1.31***	1.01, 1.62
Private Insurance	0.85*	0.72, 1.00	0.91	0.74, 1.12	0.17***	0.14, 0.21	1.51***	1.12, 1.90	-0.74***	-0.92, -0.56
Interviewed in English	1.71***	1.32, 2.21	1.91***	1.27, 2.88	-0.01	-0.07, 0.06	-0.24	-0.81, 0.32	0.47***	0.20, 0.74
Family Income <100% FPL	1.41***	1.20, 1.66	1.33***	1.10, 1.61	-0.19***	-0.23, -0.14	-2.35***	-2.84, -1.85	1.20***	0.97, 1.44
Family Income 100–200% FPL	1.26***	1.10, 1.45	1.19*	1.00, 1.42	-0.16***	-0.20, -0.12	-1.50***	-1.88, -1.12	0.78***	0.60, 0.95
Stroke	2.48*	1.14, 5.40	0.55	0.18, 1.68	-0.30	-0.73, 0.12	-2.51	-5.91, 0.90	1.79	-0.06, 3.64
AMI	1.10	0.83, 1.45	0.91	0.64, 1.30	-0.08	-0.19, 0.03	-1.10	-2.23, 0.03	0.35	-0.12, 0.83
Hyperlipidemia	1.17*	1.01, 1.35	1.51***	1.29, 1.77	-0.05*	-0.08, -0.01	-0.27	-0.62, 0.09	0.09	-0.06, 0.24
Hypothyroidism	1.60	0.97, 2.66	1.71*	1.09, 2.69	-0.15*	-0.30, -0.01	-1.63*	-3.24, -0.02	0.56	-0.09, 1.21
Hypertension	1.51***	1.31, 1.74	1.29**	1.08, 1.54	-0.14***	-0.17, -0.10	-1.00***	-1.33, -0.67	0.59***	0.44, 0.74
Urban Residence	1.14	0.99, 1.32	1.35***	1.13, 1.61	0.05**	0.01, 0.09	-0.42*	-0.82, -0.02	0.10	-0.08, 0.28
Midwest	1.23*	1.04, 1.46	1.01	0.80, 1.28	-0.02	-0.06, 0.03	-0.23	-0.66, 0.20	0.18*	0.01, 0.35
South	1.01	0.85, 1.20	1.01	0.80, 1.29	0.04	-0.01, 0.08	0.03	-0.40, 0.46	0.09	-0.10, 0.27
West	1.10	0.92, 1.32	1.02	0.80, 1.30	0.04*	0.00, 0.09	-0.09	-0.55, 0.38	0.12	-0.08, 0.32
_constant	0.03***	0.02, 0.04	0.02***	0.01, 0.03	4.27***	4.17, 4.38	50.89***	49.98, 51.81	3.03***	2.57, 3.48

* p ≤ 0.05;

** p ≤ 0.01;

*** p ≤ 0.001

We conducted stratified analyses to see whether these findings are consistent across the four different regions of the U.S. (Tables 6 and 7). We found that males in the West region had lower odds of being diagnosed with depression during and after the recession compared to pre-recession. Moreover, males in the West, Midwest, and Southern regions had better self-reported mental health during and after the recession compared to pre-recession.

**Table 6:** Stratified multivariate analyses of recession indicators (during and after) and mental health diagnoses for males.

	Depression Diagnosis (N = 38670) ref.- Before Recession				Anxiety Diagnosis (N = 38670) ref.- Before Recession			
	During		After		During		After	
	OR	95% CI	OR	95% CI	OR	95% CI	OR	95% CI
Males Stratified by Region								
Northeast	0.76	0.55, 1.05	1.17	0.86, 1.59	1.05	0.56, 1.47	0.86	0.57, 1.30
Midwest	0.90	0.69, 1.17	0.91	0.72, 1.14	0.96	0.74, 1.24	1.02	0.79, 1.32
South	0.90	0.69, 1.17	0.91	0.72, 1.14	0.96	0.74, 1.24	1.02	0.79, 1.32
West	0.76*	0.60, 0.94	0.69**	0.53, 0.91	0.86	0.60, 1.23	0.89	0.63, 1.25
Males Stratified by Family Income								
<100% FPL	0.72*	0.52, 0.99	0.99	0.74, 1.33	0.86	0.61, 1.21	0.92	0.65, 1.29
100–200% FPL	0.69**	0.52, 0.91	0.82	0.64, 1.06	1.06	0.74, 1.50	0.79	0.56, 1.12
>200% FPL	0.83*	0.71, 0.97	0.85*	0.72, 1.00	0.98	0.81, 1.19	1.09	0.90, 1.32
Males Stratified by Employment								
Employed	0.79**	0.68, 0.92	0.81*	0.69, 0.95	0.94	0.78, 1.14	1.02	0.84, 1.24
Unemployed	0.80	0.63, 1.01	1.05	0.83, 1.33	1.1	0.84, 1.44	1.00	0.77, 1.30

* p ≤ 0.05;

** p ≤ 0.01;

*** p ≤ 0.001

**Table 7:** Stratified multivariate analyses of recession indicators (during and after) and mental health indicators for males.

	Self-Reported Mental Health Score (N = 38592) ref.- Before Recession				MCS Score (N = 35713) ref.- Before Recession				Kessler Index Score (N = 35164) ref.- Before Recession			
	During		After		During		After		During		After	
	Coef.	95% CI	Coef.	95% CI	Coef.	95% CI	Coef.	95% CI	Coef.	95% CI	Coef.	95% CI
Males Stratified by Region												
Northeast	0.04	-0.06, 0.13	-0.06	-0.16, 0.04	0.27	-0.60, 1.14	0.08	-0.71, 0.88	-0.28	-0.62, 0.06	-0.16	-0.50, 0.18
Midwest	0.07*	0.01, 0.13	0.07*	0.01, 0.14	0.28	-0.33, 0.89	0.40	-0.24, 1.04	-0.12	-0.41, 0.16	-0.27	-0.55, 0.01
South	0.07*	0.01, 0.13	0.07*	0.01, 0.14	0.28	-0.33, 0.89	0.40	-0.24, 1.04	-0.12	-0.41, 0.16	-0.27	-0.55, 0.01
West	0.09**	0.03, 0.15	0.08*	0.01, 0.15	-0.44	-1.07, 0.18	0.30	-0.35, 0.94	0.21	-0.07, 0.48	-0.23	-0.52, 0.06
Males Stratified by Family Income												
<100% FPL	0.17**	0.07, 0.27	0.15**	0.05, 0.25	0.65	-0.43, 1.74	1.54**	0.43, 2.65	-0.22	-0.73, 0.30	-0.60*	-1.12, -0.08
100–200% FPL	0.05	-0.02, 0.13	0.08	0.00, 0.15	0.14	-0.68, 0.96	0.71	-0.17, 1.60	-0.10	-0.48, 0.28	-0.50*	-0.90, -0.11
>200% FPL	0.04*	0.00, 0.08	0.01	-0.03, 0.06	0.06	-0.30, 0.41	0.02	-0.34, 0.39	-0.01	-0.16, 0.14	-0.08	-0.24, 0.07
Males Stratified by Employment												
Employed	0.04*	0.01, 0.08	0.03	-0.01, 0.07	0.03	-0.28, 0.35	0.13	-0.20, 0.46	0.00	-0.13, 0.13	-0.13	-0.27, 0.01
Unemployed	0.12*	0.02, 0.23	0.07	-0.03, 0.17	0.63	-0.53, 1.78	1.04	-0.10, 2.18	-0.31	-0.85, 0.23	-0.52*	-1.02, -0.01
Males Stratified by Health Service Use												
Users	0.14*	0.02, 0.26	0.05	-0.06, 0.16	0.01	0.00, 0.02	0.01	0.00, 0.02	-0.36	-0.96, 0.24	-0.36	-0.98, 0.25
Non-users	0.02	-0.13, 0.17	-0.16*	-0.31, -0.01	0.00	-0.02, 0.02	0.01	-0.01, 0.03	0.13	-0.66, 0.91	-0.14	-0.93, 0.66

* p ≤ 0.05;

** p ≤ 0.01;

*** p ≤ 0.001

We also explored whether our findings differed by employment status by conducting separate analyses on unemployed versus employed males (Tables 6 and 7). We found that among the unemployed, males had better self-reported mental health during the recession and lower scores on the Kessler scale after the recession compared to pre-recession. On the other hand, among the employed, males had lower odds of being diagnosed with depression during and after the recession and better self-reported mental health during the recession compared to pre-recession.

Stratification analyses by income among males showed that people with low household income had lower odds of depression diagnoses during the recession, better self-reported mental health during and after the recession, higher mental health summary scores after the recession, and lower scores on the Kessler scale after the recession, in comparison to pre-recession (Tables 6 and 7). People with middle household income had lower odds of depression diagnoses during the recession, and better self-reported mental health and lower scores on the Kessler scale after the recession, in comparison to pre-recession. Finally, people with high household income had lower odds of depression diagnoses during and after the recession, and better self-reported mental health during the recession, in comparison to pre-recession.

We also conducted stratified analyses by health care utilization (use of at least one service) among those who were diagnosed with anxiety or depression (Table 7). We found that among males, non-users of health services had worse self-reported mental health after the recession; however, users of health services had better self-reported mental health during the recession.

## Discussion

In this large nationally representative study of the U.S. population, we found that both males and females were less likely to be diagnosed with depression during and after the recession compared to pre-recession; however, females were more likely to be diagnosed with anxiety after the recession compared to pre-recession. In general, past studies suggest higher vulnerability of men to the negative mental health consequences of economic recessions [16]. However, this may not be the case anymore given the increasingly high labor force participation rate of women [37] and work becoming an important part of the self-identity of the majority of women [38]. Another potential explanation is the consistent evidence in the literature that females are twice more likely to have an anxiety disorder than males [39]. These general differences in anxiety among males and females may have persisted despite men losing disproportionately more jobs during the recession than women.

The stratified analyses showed that the higher odds of anxiety diagnoses among females after the recession were specifically prominent among specific subgroups of females: those who lived in the Northeast or the Midwest, the unemployed, and those with low household income. These findings alert us to important disparities by region of residence, employment status, and income that are specific to females. Thus, policymakers should consider targeting these vulnerable subgroups of women in the wake of a recession.

The differences in anxiety and depression diagnoses and self-reported measures of mental health during and post-recession are above and beyond the effects of unemployment, which are controlled in the analyses. Moreover, the findings were consistent when we stratified by U.S. region, employment, and income. Males and females had lower odds of being diagnosed with depression during and after the recession compared to pre-recession years. In addition, females had better self-reported mental health scores during the recession and males had better mental health scores during and after the recession, compared to pre-recession. These generally positive mental health outcomes are in contrast to our hypothesis that people experience worse mental health outcomes during the recession. One possible explanation is what some researchers have hypothesized and shown that during economic downturns, there is more leisure time to spend on family, friends, and exercise [9, 40–42], which may decrease the likelihood of depression. Moreover, other researchers have shown that the impact of recessions on mental health is context-dependent, with negative impacts more apparent in low-income and middle-income countries while some affluent countries providing supports and services that help their citizens escape the negative consequences of recessions [43].

Overall, these associations could partly be due to social protection programs in the U.S. such as unemployment compensation, debt relief programs, and social welfare. Moreover, the passage of the Mental Health Parity and Addiction Equity Act of 2008 [44] may have facilitated access to mental health care in a timely fashion which may have prevented serious mental illnesses. As seen from the stratified analyses by health services use, males and females who were diagnosed with anxiety or depression and did not receive any health services had worse self-reported mental health during and after the recession compared to pre-recession. It is foreseeable that the Affordable Care Act will help these disadvantaged groups that have no access to health services with its mandate for state-insurance exchanges to have a base-level package that includes mental health coverage, its mandate for insurers to cover depression screening among other preventive services for free, and expansions of eligibility in Medicaid programs.

### Limitations

The results of this study should be interpreted in light of its limitations. First, we were limited to cross sectional analyses due to the MEPS survey design, which precluded causal inferences of the study results. Future studies should use data that follow the same people over time to facilitate longitudinal analyses. However, it is important to mention that the samples during and after the recession did not differ from those before the recession in terms of racial/ethnic composition, marital status, language of the interview, urban versus rural residence, or by geographic location and thus the demographic characteristics of the cohorts studied were similar. Second, it is possible that the trends in mental health outcomes before, during and after the recession were due to other unobserved factors such as geographic variation in unemployment rates, temporal changes, or implementation of different state and local policies to alleviate the economic impact of the recession on people’s lives. However, the findings were generally consistent across the different mental health measures and we controlled for the employment status of individuals. Moreover, the findings were consistent in the stratified analyses by U.S. region, income, and employment status. A third limitation pertains to our data not including information about economic suicides. The rate of increase in suicides in the U.S. has been shown to have accelerated between 2007 and 2010 [45]; thus, future studies should examine the association of the economic recession with an additional indicator of mental health, suicide rates. Fourth, while we found statistically significant associations between the recession and different mental health outcomes, these associations were small and may not be clinically significant. It may be that clinically significant effects of the recession on mental health take longer time periods to develop than the periods observed in our study. Fifth, we acknowledge that the five mental health outcomes examined in our study were self-reported and not clinical diagnoses; thus they may suffer from respondent bias. Also, despite the fact that self-reported depression and anxiety diagnoses were verified by physicians, there is potential for recall bias. Finally, defining recession periods and their effects on health are difficult in general and we did not have macroeconomic measures such as unemployment rates in our data. Future research should examine state specific unemployment rates to examine the impact of economic recessions on mental health outcomes.

## Conclusion

This study examined and compared five mental health outcome measures in 2005–2006, 2008–2009, and 2010–2011 separately by gender, using a nationally representative dataset. We found that both females and males reported better mental health status, and were less likely to be diagnosed with depression during and after the recession compared to pre-recession. However, females were more likely to be diagnosed with anxiety after the recession. Future research should examine the reasons behind lower diagnoses of depression, and whether they signify less visits to mental health providers or increased social support in communities and more time for exercise and leisure activities. While policymakers may interpret these results as positive in general, our findings that among females who lived in the Northeast or the Midwest, were unemployed, or had low household income, there were higher anxiety diagnoses after the recession, and that among males and females who did not receive mental health care there were worse mental health outcomes during and after the recession raises questions regarding the impact of the recession on the mental health of specific vulnerable population groups. Policymakers should invest in labor market programs that provide group psychological support for the unemployed and reintegrate workers in jobs and consider other social policies such as debt relief programs to help people cope with the recession and reduce the anxiety and other mental health consequences associated with the recession.
